# Cervical Cancer Cell Supernatants Induce a Phenotypic Switch from U937-Derived Macrophage-Activated M1 State into M2-Like Suppressor Phenotype with Change in Toll-Like Receptor Profile

**DOI:** 10.1155/2014/683068

**Published:** 2014-09-21

**Authors:** Karina Sánchez-Reyes, Alejandro Bravo-Cuellar, Georgina Hernández-Flores, José Manuel Lerma-Díaz, Luis Felipe Jave-Suárez, Paulina Gómez-Lomelí, Ruth de Celis, Adriana Aguilar-Lemarroy, Jorge Ramiro Domínguez-Rodríguez, Pablo Cesar Ortiz-Lazareno

**Affiliations:** ^1^División de Inmunología, Centro de Investigación Biomédica de Occidente (CIBO), Instituto Mexicano del Seguro Social (IMSS), Sierra Mojada 800, Col. Independencia, 44340 Guadalajara, JAL, Mexico; ^2^Programa de Doctorado en Ciencias Biomédicas Orientación Inmunología, Centro Universitario de Ciencias de la Salud (CUCS), Universidad de Guadalajara, 44340 Guadalajara, JAL, Mexico; ^3^Departamento de Ciencias de la Salud, Centro Universitario de los Altos, Universidad de Guadalajara, Tepatitlán de Morelos, 47600 Guadalajara, JAL, Mexico; ^4^Departamento de Farmacobiología, Centro Universitario de Ciencias Exactas e Ingeniería, Universidad de Guadalajara, 44430 Guadalajara, JAL, Mexico

## Abstract

Cervical cancer (CC) is the second most common cancer among women worldwide. Infection with human papillomavirus (HPV) is the main risk factor for developing CC. Macrophages are important immune effector cells; they can be differentiated into two phenotypes, identified as M1 (classically activated) and M2 (alternatively activated). Macrophage polarization exerts profound effects on the Toll-like receptor (TLR) profile. In this study, we evaluated whether the supernatant of human CC cells HeLa, SiHa, and C-33A induces a shift of M1 macrophage toward M2 macrophage in U937-derived macrophages. *Results*. The results showed that soluble factors secreted by CC cells induce a change in the immunophenotype of macrophages from macrophage M1 into macrophage M2. U937-derived macrophages M1 released proinflammatory cytokines and nitric oxide; however, when these cells were treated with the supernatant of CC cell lines, we observed a turnover of M1 toward M2. These cells increased CD163 and IL-10 expression. The expression of TLR-3, -7, and -9 is increased when the macrophages were treated with the supernatant of CC cells. *Conclusions*. Our result strongly suggests that CC cells may, through the secretion of soluble factors, induce a change of immunophenotype M1 into M2 macrophages.

## 1. Background

Cervical cancer (CC) is the second most common cancer among women worldwide [1]. Persistent infection with high-risk human papillomaviruses (HPV), such as HPV-16, -18, and -45, comprises the most important factors for the development of CC [2–4]. The first line of defense against HPV infection in early infection is the innate immune system, which plays a crucial role in viral clearance [5]. Macrophages have long been considered as important immune effector cells involved in primary response to pathogens, normal tissue homeostasis, the presentation of foreign and self-antigens following infection or injury, resolution of inflammation and wound healing, and engaging bidirectional interaction with lymphocytes and other innate lymphoid-cell subsets [6–8]. Macrophages originate from blood monocytes; they differentiate into distinct macrophage types depending on the microenvironment. Because T_H_1 cytokines, such as interferon-gamma (IFN-*γ*) and bacterial cell wall-derived lipopolysaccharides (LPS), polarize the macrophage toward the M1 phenotype, characterized by their ability to release proinflammatory cytokines such as tumor necrosis factor-alpha (TNF-*α*), Interleukin- (IL-) 1*β*, IL-6, IL-12, IL-23, reactive nitrogen and oxygen intermediates, higher expression of major histocompatibility complex class II, efficient antigen presentation, and microbicidal and tumoricidal activity. On the other hand, T_H_2 cytokines IL-4, IL-13, and IL-10, as well as glucocorticoids, give rise to M2 macrophages, a phenotype characterized by low expression of IL-12 and high expression of IL-10, transforming growth factor-beta (TGF-*β*), angiogenic factors, high expression of scavenger and mannose and galactose receptors CD163 and CD206, respectively, deficient capacity as antigen-presenting cells, low antitumor activity, and an increase in the ability to support angiogenesis and tissue remodeling, facilitating tumor growth and invasion [8, 9].

Macrophage phenotype and activation are regulated by cytokines and also by pattern recognition receptors that include the Toll-like receptors (TLR). TLR play a critical role in innate immunity by recognizing pathogen-associated molecular patterns (PAMP) and endogenous damage-associated molecular patterns (DAMP), such as ligands, to induce an innate immune response. Ten functional TLR have been identified in humans and their specific ligands, including LPS detected by TLR-4, bacterial lipoproteins, and lipoteichoic acids by TLR-2, flagellin detected by TLR-5, the unmethylated CpG DNA of bacteria and viruses detected by TLR-9, double-stranded RNA detected by TLR-3, and single-stranded viral RNA detected by TLR-7 [10–12]. DAMP include high motility group box-1 (HMGB1), heat shock protein (HSP), self-double-stranded RNA, and single-stranded RNA. TLR ligation and signaling lead to the expression of effector molecules, such as proinflammatory cytokines and interferon (IFN) [13]. Several publications have demonstrated that TLR also play an important role in tissue repair and inflammation-induced tissue damage. In this respect, it was published that the macrophage count increased linearly with disease progression in CC [14]. The role of tumor-associated macrophages (TAM) remains controversial; in colorectal tumors, TAM are proinflammatory and play an antitumor role [15]. However, in a variety of tumors such as breast, prostate, ovarian, cervical, and lung carcinoma and cutaneous melanoma, TAM were considered as anti-inflammatory and correlated with poor prognosis [7]. Many observations indicate that TAM express several functions associated with the M2 macrophage phenotype, including promotion of angiogenesis, matrix remodeling, and suppression of adaptative immunity [16]. Recently, it was demonstrated that carcinoma cells hamper monocyte-to-dendritic cell differentiation and change their differentiation into M2 macrophages [17]. In this study, we employed the supernatant of CC cell lines HeLa, SiHa, and C-33A to evaluate the effect on the modulation of the change of immunophenotypic M1 into M2 in U937-derived macrophages activated with LPS, as well as studying the TLR-3, -7, and -9 expression in these cells.

## 2. Methods

### 2.1. Cell Lines

HeLa (HPV-18+) and SiHa (HPV-16+) cells, C-33A (HPV–) CC cells, and nontumorigenic human keratinocyte cell line HaCaT were kindly provided by Dr. Boukamp (DKFZH, Heidelberg, Germany). The presence of the human papillomavirus (HPV) type was confirmed by the linear array genotyping test (Roche). U937 cells were obtained from the American Type Culture Collection (ATCC CRL-1593.2; Manassas, VA, USA). CC cells (HeLa, SiHa, and C-33A) were maintained* in vitro* propagated in Dulbecco's modified Eagle's culture medium (DMEM; GIBCO Invitrogen Corp., Carlsbad, CA, USA) with 10% heat-inactivated Fetal bovine serum (FBS; GIBCO Invitrogen Corp.), and U937 cells were maintained* in vitro* propagated in Roswell Park Memorial Institute- (RPMI-) 1640 culture medium (GIBCO Invitrogen Corp.) and FBS at a final concentration of 10% (GIBCO Invitrogen Corp.); both media were supplemented with 1X L-glutamine (at a 2 mM final concentration; GIBCO Invitrogen Corp.) and antibiotics (Penicillin/Streptomycin; GIBCO Invitrogen Corp.). These media will be referred to as DMEM-S and RPMI-S. Cells were incubated at 37°C in a humidified atmosphere containing 95% air and 5% CO_2_.

### 2.2. Supernatant of CC Cell Lines

CC cell lines HeLa, SiHa, C-33A, and HaCaT were grown in flasks at 80–90% confluence and harvested with trypsin. After that, 500,000 HaCaT cells or 100,000 HeLa, SiHa, or C-33A cells were plated on 2 mL of DMEM-S on 6-well culture plates. Cells were incubated at 37°C in a humidified atmosphere containing 95% air and 5% CO_2_ for 5 days. Afterward, the cultured supernatant of these cell lines was collected and stored at –80°C until needed for cytokine analysis or for use in the culture of U937-derived macrophages in the corresponding experimental groups.

### 2.3. Induction of U937 Differentiation and Activation

U937 cell lines (1 × 10^6^ cells) were differentiated into macrophages on a 12-well tissue-culture plate containing 2 mL of the RPMI-1640 medium in the presence 200 nM of Phorbol myristate acetate (PMA) for 3 days [18]. After incubation, nonattached cells were removed by aspiration, and adherent cells were washed with PBS three times. For inducing macrophage activation (M1) [19], differentiated cells were treated with 100 ng/mL of Lipopolysaccharide (LPS) for 24 h; afterward, the cells were washed with PBS thoroughly four times to completely remove the LPS.

### 2.4. Experimental Conditions

U937 cells differentiated into macrophages and activated with LPS (M1 macrophages) were treated or not with the supernatant of HeLa, SiHa, C-33A, and HaCaT cells at a final concentration of 30% of the total volume. Then, the cells were incubated for 7 days in a humidified atmosphere containing 95% air and 5% CO_2_. Next, the supernatants of these cultures were collected and stored at –80°C until cytokine profile analysis and nitric oxide (NO) assessment. Following this, the cells were detached with accutase solution (BD Biosciences, San Jose, CA, USA) and stained for assessment of CD163 and TLR by flow cytometry (FC) analysis. Dexamethasone (DEX) at a final concentration of 200 ng/mL was used as positive control for induction of M2 macrophages [20]. The supernatant from the nontumorigenic cell line HaCaT was used as negative control.

### 2.5. Assessment of CD163 and TLR by Flow Cytometry

Expression of CD163 and TLR was assessed by FC. Briefly, all cells in the different experimental groups were detached, washed twice with PBS, and resuspended in PBS. Then, we blocked human Fc receptors (FcR) using Fc Receptor Blocking Solution (BioLegend, San Diego, CA, USA) for 10 min prior to staining with antibodies. After that, the cells were incubated with antihuman CD163-APC antibody (BioLegend) for 30 min at 4°C. Subsequently, the cells were washed and permeabilized with permeabilization buffer 1X (BioLegend), and we added antihuman TLR-3-Fluorescein isothiocynate (FITC) antibody (Abcam, Cambridge, UK) or antihuman TLR-7-FITC antibody (Abcam) or antihuman TLR-9-FITC antibody (Abcam) for 30 min at 4°C. Then, the cells were washed with PBS, fixed with paraformaldehyde 1%, and analyzed by FC. An appropriate isotype control was utilized to adjust for background fluorescence, and results are reported as the % of expression or as the geometric mean fluorescence intensity (MFI). For each sample, at least 10,000 events were acquired in a FACSAria I cell sorter (BD Biosciences). Data were processed with FACSDiva software (BD Biosciences).

### 2.6. Assessment of Cytokines by Flow Cytometry

Supernatant collected in the different experimental groups was analyzed to determine the cytokine profile concentration. We used the Human Th1/Th2/Th9/Th17/Th22 13 plex FlowCytomix Multiplex (eBioscience, San Diego, CA, USA) to analyze IL-1*β*, IL-2, IL-5, IL-6, IL-10, IL-4, IL-9, IL-22, IL-12p70, IL-13, IL-17A, Interferon-gamma (IFN-*γ*), and tumor necrosis factor-alpha (TNF-*α*) cytokines, according to the manufacturer's instructions. Briefly, 25 *μ*L of supernatant of all of the experimental groups or standard mixture dilutions or assay buffer or bead mixture was added to the designated tubes; afterward, 50 *μ*L of the biotin-conjugated mixture was added, and tubes were incubated for 2 h and were protected from exposure to light at room temperature. Subsequently, the tubes were washed twice with assay buffer; then, we added 50 *μ*L de Streptavidin-PE solution to all tubes and incubated these for 1 h, protected from light, at room temperature. Finally, all tubes were washed twice with assay buffer; then we added 500 *μ*L of assay buffer to each tube and samples were acquired in a FACSAria I cell sorter (BD Bioscience). The results were analyzed with FlowCytomix Pro 3.0 Overview software (eBiosciences) and were expressed in pg/mL.

### 2.7. Measurement of Nitric Oxide by Colorimetric Assay

Supernatant of all experimental groups was collected to determine NO concentration by a colorimetric assay (Total Nitric Oxide and Nitrate/Nitrite; R&D Systems, Minneapolis, MN, USA). The assay was performed as described by the manufacturer. Briefly, for the nitrite assay, 50 *μ*L of sample was combined in a well with reaction diluent and Griess reagents I and II; after 10 min of incubation, the optical density (OD) was measured using a microplate reader (Synergy HT Multi-Mode Microplate Reader; Bio Tek Instruments, Inc., Winooski, VT, USA) at 540 nm (wavelength correction at 690 nm). For the nitrate reduction assay, 50 *μ*L of sample was mixed with Nicotinamide adenine dinucleotide (NADH) and nitrate reductase and, after incubating this for 30 min at room temperature, we added Griess reagents I and II. Finally, after 10 min at room temperature, OD was determined at 540 nm (wavelength correction at 690 nm). Results are expressed in *μ*mol/L.

### 2.8. Statistical Analysis

The data obtained are shown as mean ± standard deviations (SD) of the values of at least three independent experiments, carried out in triplicate. Comparisons among groups were performed with Student's  *t*-test. Only values of  *P* < 0.05 were considered significant.

## 3. Results

### 3.1. Supernatant of Cervical Cancer Cell Lines HeLa, SiHa, and C-33A Positively Regulates the Expression of CD163 in U937-Derived Macrophages Activated with LPS

**Figure 1:**
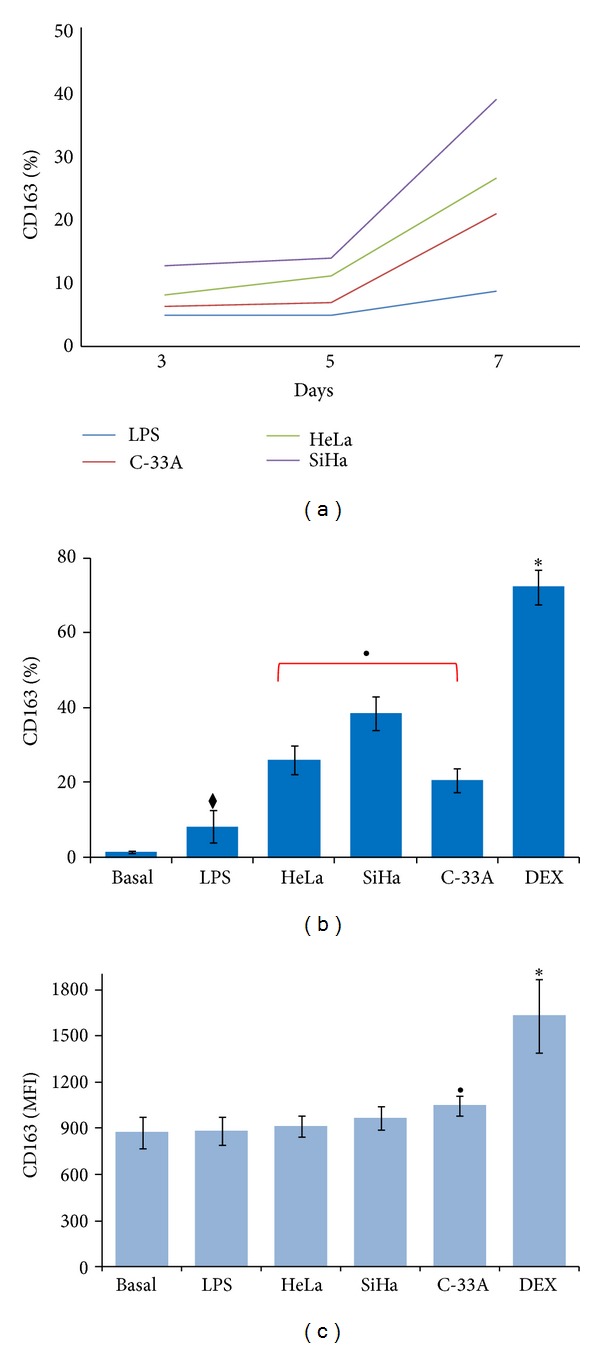
Supernatant of HeLa, SiHa, and C-33A induces CD163 upregulation in U937-derived macrophages activated with lipopolysaccharides (LPS). U937 cells were differentiated into macrophages and treated with LPS for 24 h. Supernatants of cervical cancer (CC) cells were added to a final concentration of 30% in corresponding groups, and the cells were incubated for 7 days at 37°C in a humid atmosphere containing 5% CO_2_ and 95% air in Roswell Park Memorial Institute- (RPMI-) S medium. Then, the expression of CD163 was analyzed by flow cytometry (FC). Expression of CD163 in U937-derived macrophages activated with LPS and treated with supernatant of HeLa, SiHa, and C-33A cells during 3, 5, and 7 days (a). Percentage of CD163 expression in U937-derived macrophages activated with LPS and treated with supernatant of HeLa, SiHa, and C-33A during 7 days (b). Values of geometric mean fluorescence intensity (MFI) for CD163 expression in the different experimental groups (c). Dexamethasone (DEX) was used as positive control in the induction of CD163 expression. Results are shown as % and MFI and represent the mean ± standard deviation (SD) of three independent experiments performed in triplicate. Statistical analysis, Student's *t*-test. ^◆^ *P* < 0.05 LPS versus Basal group. ^•^ *P* < 0.05 HeLa, SiHa, and C-33A groups versus Basal and LPS groups. *P < 0.0001 DEX versus all groups.

CD163 is a marker restricted to linage monocyte-macrophages, and it has been suggested that it is principally expressed in macrophages with an immunosuppressive phenotype (M2 macrophage) [21]. We first investigated whether the supernatant of CC cell lines HeLa, SiHa, and C-33A induces the expression of CD163 in U937-derived macrophages activated with LPS (M1 macrophages, which will be designated as the LPS group). Previous models* in vitro* using cell lines with regard to monocyte differentiation into M2 macrophages were carried out from 48 h to 7 days [17, 22]. Based on this, we performed expression kinetics for the CD163 receptor, incubating the LPS-activated macrophages in the presence or not of supernatants (at final concentrations of 30%) of HeLa (HeLa group), SiHa (SiHa group), and C-33A (C-33A group) during 3, 5, and 7 days. In Figure 1(a), we can observe that the increase in CD163 expression on day 3 is very close to that of the LPS control group. Afterward, we observed an increase that was more evident at day 7; at this point (Figure 1(b)), we observed a significant increase of CD163 expression on incubation day 7, with the LPS exclusively treated group as 100%. In HeLa group, we observed 26.3% positive cells, which represents an increase of Δ% = 217 (*P* < 0.05); this increase was most important in SiHa group (38.7% positive cells; Δ% = 350; *P* < 0.05) and, to a lesser degree, when the cells were incubated with supernatant of HPV-free cells, C-33A; Δ% = 142 (20.8%); *P* < 0.05. Finally, the Dexamethasone (DEX) positive control group reached the highest values of CD163 positive cells 70.1%; Δ% = 743; (*P* < 0.0001).

We also studied the surface-cell expression density of the CD163 receptor. In Figure 1(c), we are able to observe that MFI are practically not modified in relation to either Basal, LPS, HeLa or SiHa cells. In contrast, C-33A cells exhibited an increase of MFI in comparison with the remaining groups (*P* < 0.05). As expected, the DEX positive control group also reached higher values (*P* < 0.0001). We also studied whether the supernatant from nontumorigenic cell line HaCaT induced CD163 expression on U937-derived macrophages. As expected, the supernatant from nontumorigenic cells did not induce a significant increase in CD163 expression under our experimental conditions (data not shown). Taken together, our results strongly suggestthat supernatant of CC cells induced positive regulation in the percentage of cells expressing the M2 phenotype.

### 3.2. Release of IL-6, IL-13, IL-2, and IL-12 in Cervical Cancer Cell Lines

Once it was demonstrated that CD163 expression is strongly upregulated in U937-derived macrophages activated with LPS by means of the effect of supernatant from HeLa (HPV-18+), SiHa (HPV-16+), and, to a lesser degree, C-33A (HPV−) cells, we determined by FC, the cytokine profile present in the supernatant of CC cells [21].

**Figure 2:**
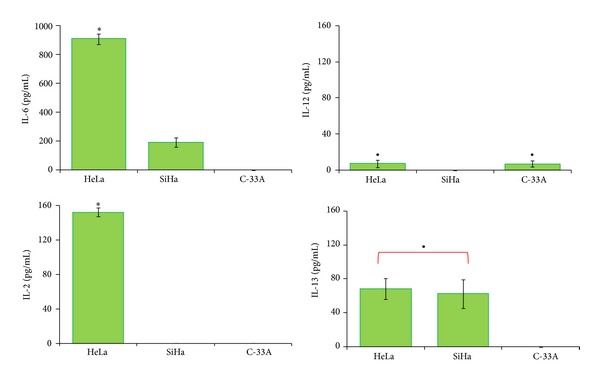
Cytokine profile production in HeLa, SiHa, and C-33A cervical cancer (CC) cells. CC cell lines were cultured in flask at 80–90% confluence and harvested, and subsequently 100,000 cells were plated on 6-well culture plates and cultured for 5 days. Supernatant was collected and concentration of cytokines was determined by flow cytometry (FC) using a cytometric bead array (CBA) kit. The results represent the mean ± standard deviation (SD) of concentrations in pg/mL for each cytokine. Statistical analysis, Student's *t*-test. *P < 0.0001 HeLa versus SiHa and C-33A groups; ^•^ *P* < 0.05 HeLa and SiHa versus C-33A group, or HeLa and C-33A versus SiHa group.

In Figure 2, we can observe that HeLa cells release the following in the supernatant: IL-6 (909.00 ± 10.2 pg/mL; *P* < 0.0001), and SiHa cells demonstrated a less important release of IL-6 (192.30 ± 5.3 pg/mL), which was not detectable in C-33A cells. Secretion of IL-12 in HeLa was 7.5 ± 4.2 pg/mL, and in SiHa cells, IL-12 was not detectable (*P* < 0.05), while in the case of C-33A, the supernatant concentration of IL-12 was 7.4 ± 3.4 pg/mL. Only IL-2 was detectable in supernatants from HeLa cultures (152.2 ± 5.0 pg/mL; *P* < 0.0001), and IL-13 was found only in supernatants from HeLa and SiHa cells (68.3 ± 12.5 pg/mL and 62.5 ± 16.9, resp.; *P* < 0.05).

### 3.3. U937-Derived Macrophages Activated with LPS Released Proinflammatory Cytokines and Supernatant of Cervical Cancer Cells Induce a Decrease in These Cytokines with an Increase in IL-10 Production

**Figure 3:**
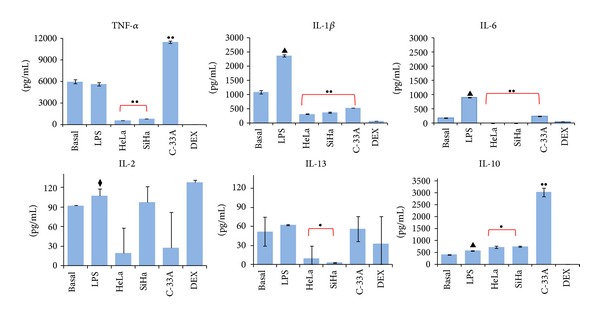
Supernatant of cervical cancer (CC) cells induces a decrease in proinflammatory cytokines and an increase in Interleukin-10 (IL-10). U937-derived macrophages activated with lipopolysaccharides (LPS) were treated or not with supernatant of HeLa, SiHa, and C-33A or Dexamethasone (DEX) for 7 days. After that, supernatant of this culture was collected and cytokine concentrations were measured by flow cytometry (FC) using a cytometric bead array (CBA) kit. The results represent the average concentrations in pg/mL for each cytokine and represent the mean ± standard deviation (SD). Statistical analysis, Student's *t*-test. ^••^ *P* < 0.0001 Basal and LPS versus HeLa, SiHa, and C-33A groups. ^•^ *P* < 0.05 Basal and LPS groups versus SiHa and HeLa groups. ^▲^ *P* < 0.0001 LPS versus Basal group. ^◆^ *P* < 0.05 LPS versus Basal group.

Communication and interaction among the cells of the immune system depend on molecules such as cytokines. Thus, it was in our interest to investigate whether supernatant of HeLa, SiHa, and C-33A CC cells could induce change in the cytokine profile secreted by U937-derived macrophages activated with LPS (M1 macrophages).  Figure 3 depicts the cytokines produced by the different experimental groups. We can observe high production of TNF-*α* in the Basal group (5,985 ± 250 pg/mL) and in the LPS group (5,620  ± 220.1 pg/mL); however, secretion of TNF-*α* was prevented by the presence of supernatant from HeLa cultures (~10 times lower), and this was ~7 times lower in the case of SiHa cells in comparison with the LPS and Basal groups (*P* < 0.0001). In contraposition, the C-33A supernatant induces ~2 times higher TNF-*α* production in comparison with basal values and the LPS group. Secretion of IL-1*β* in macrophages activated with LPS was increased with respect to the Basal group (2,371 ± 35.0 pg/mL versus 1,096 ± 61.5 pg/mL, resp.; *P* < 0.0001). However, secretion of IL-1*β* was diminished in macrophages cultured with HeLa supernatants ~10 times, in the case of SiHa ~6 times, and in the case of C-33A supernatant 4 times in comparison with basal and with macrophages activated with LPS (*P* < 0.0001). Also in Figure 3, we are able to observe that IL-6 was secreted by nonactivated macrophages and reached 174.7 ± 9.7 pg/mL, and its secretion, as expected, increased about 5 times in macrophages activated with LPS (904.9 ± 8.0 pg/mL; *P* < 0.0001). Amazingly, the release of IL-6 was completely inhibited in the presence of HeLa and SiHa supernatants, but not in the case of macrophages cultured in the presence of C-33A supernatant, in which it was possible to detect IL-6 (232.7 ± 8.7 pg/mL) at a concentration ~4 times lower in comparison with that of the LPS group (*P* < 0.0001). In the case of IL-2, production by macrophages reached basal values of 91.4 ± 1.4 pg/mL, and, after LPS stimulation, this was 106.9 ± 10.41 pg/mL; this secretion decreased ~4 times in the presence of HeLa and C-33A supernatants (19.1 ± 38.3 and 27.4 ± 54.7 pg/mL, resp.; *P* < 0.05). However, with the SiHa cell supernatant, the concentration of IL-2 was only slightly lower (96.58 ± 24.4 pg/mL) with respect to that of macrophages activated with LPS. The basal level of IL-13 was 51.9 ± 22.4 pg/mL, and, when the macrophages were stimulated with LPS (62.3 ± 0.8 pg/mL), this secretion was prevented by the presence of the HeLa supernatant by ~6 times (9.7 ± 19.5 pg/mL; *P* < 0.05) and, surprisingly, was ~20 times lower in the case of SiHa cell supernatants (3.0 ± 0.6 pg/mL; *P* < 0.05). In the presence of the C-33A supernatant, we only observed a slight modification (55.75 ± 19.78 pg/mL) in contrast with LPS-activated macrophages. Anti-inflammatory cytokine IL-10 was released by nonactivated macrophages and M1 macrophages activated with LPS (404.8 ± 6.9 pg/mL and 571.1 ± 8.5 pg/mL, resp.; *P* < 0.0001). Interestingly, when macrophages were cultured with HeLa cell supernatant, we observed Δ% = 25.6 (*P* < 0.05) in comparison with that of the LPS group. An increase of Δ% = 32.0 was observed when the macrophages were cultured with SiHa supernatant (*P* < 0.05); however, the most significant increase of IL-10 was noted with C-33A cells, where Δ% = 424 was observed in comparison with basal and M1 macrophages activated with LPS (*P* < 0.0001). Proinflammatory IL secretion was practically not observed in the control culture treated with DEX. Taken together, these results strongly suggest that U937-derived macrophages activated with LPS possess a proinflammatory profile that changes into the M2 phenotype in the presence of supernatant from CC cells.

### 3.4. Supernatant of Cervical Cancer Cells Induces a Decrease in Nitric Oxide Production in U937-Derived Macrophages Activated with LPS

**Figure 4:**
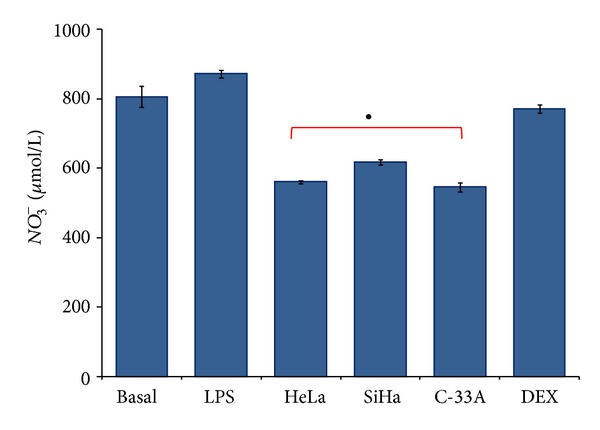
Supernatant of cervical cancer (CC) cells induces a decrease in Nitric oxide (NO) in U937-derived macrophages activated with Lipopolysaccharides (LPS). U937-derived macrophages were activated with LPS and treated or not with supernatant of HeLa, SiHa, and C-33A cells or Dexamethasone (DEX) for 7 days. After culture, supernatant was collected. Quantification of NO in the form of Nitrate (NO_3_^−^) was determined in the supernatant of all experimental groups by colorimetric assay. The results represent average concentrations in *μ*mol/L. Values represent mean ± standard deviations (SD). Statistical analysis, Student's  *t*-test. ^•^ *P* < 0.05 LPS and Basal versus HeLa, SiHa, and C-33A groups.

M1 macrophages produce high levels of NO, which is essential for antimicrobial and antitumoral activities. We studied whether HeLa, SiHa, and C-33A cell supernatant induced a modification in NO production by means of U937-derived macrophages activated with LPS. In Figure 4, we can observe a high production of NO (806.5 ± 30.5 *μ*mol/L, Basal group, and 872.3 ± 10.80 *μ*mol/L, LPS group); however, NO production decreased when macrophages activated with LPS were reincubated in the presence of HeLa, SiHa, and C-33A cell supernatant 561.2 ± 4.4, 618.3 ± 7.6, and 546.0 ± 13.3 *μ*mol/L (Δ% = −35.0, −25.0, and −37.0%, resp.) in contrast with the LPS group (*P* < 0.05). These results indicate that CC cells can interfere with NO production in macrophages. In the DEX group, a decrease in NO production was observed in comparison with that of the LPS group.

### 3.5. HeLa, SiHa, and C-33A Supernatant Induces Change in TLR Profile in U937-Derived Macrophages Activated with LPS

**Figure 5:**
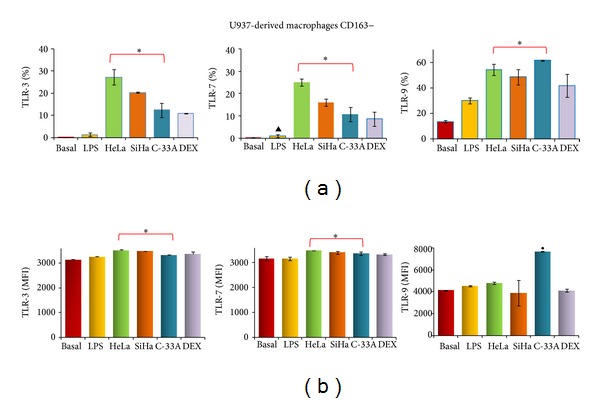
Toll-like (TLR) -3, -7, and -9 expression in M1 macrophages treated with the supernatant of cervical cancer (CC) cells. U937-derived macrophages were activated with lipopolysaccharides (LPS) and treated or not with supernatant of HeLa, SiHa, and C-33A cells or Dexamethasone (DEX) for 7 days. After that, expression of CD163 and TLR-3, -7, and -9 was analyzed by flow cytometry (FC). Percentage of TLR-3, -7, and -9 expression in CD163– U937-derived macrophages activated with LPS (a). Geometric mean fluorescence intensity (MFI) values for TLR-3, -7, and -9 in CD163− U937-derived macrophages activated with LPS (b). Results are shown as % and MFI and represent the mean ± standard deviations (SD) of three independent experiments performed in triplicate. Statistical analysis, Student's *t*-test. *P < 0.05 Basal and LPS versus HeLa, SiHa, and C-33A groups. ^•^ *P* < 0.05 C33-A versus Basal and LPS groups. ^▲^ *P* < 0.05 LPS versus Basal group.

Intracellular TLR-3, -7, and -9 recognize viral structures and mediate an antiviral response via IFN. Thus, we analyzed the expression of TLR-3, -7, and -9 in all experimental groups with either CD163+ or CD163− in U937-derived macrophages activated with LPS.  Figure 5(a) illustrates TLR-3, -7, and -9 expression in M1 macrophages (CD163–). Interestingly, TLR-3 and TLR-7 are practically not expressed in macrophages stimulated with LPS (1.3 ± 0.8 and 1.1 ± 0.7%, resp.), but when these cells were exposed to the supernatants of HeLa and SiHa cancer cells, we observed a significant increase in TLR expression as follows: in the HeLa group 27.4 ± 3.4% of TLR-3, 25 ± 1.6% of TLR-7; in the SiHa group 20.4 ± 0.2% of TLR-3 and 16 ± 1.6% of TLR-7, and, although expression of TLR in C-33A was increased (12.5 ± 3.1 for TLR-3 and 10.7 ± 3.1% for TLR-7), this was to a lesser degree than the increase observed in cell lines infected with HPV. Macrophages activated with LPS display a percentage of expression for TLR-9 of 30.1 ± 2.4%, and this was significantly increased in the C-33A and HeLa groups (61.5 and 54.2%, resp.; *P* < 0.05). The DEX group induced an increase in TLR-3 and TLR-7 in comparison with the LPS group.

It is important to consider the density of expression of TLR in experimental groups.  Figure 5(b) depicts that HeLa, SiHa, and C-33A groups induced an increase in MFI for TLR-3 and TLR-7 in M1 macrophages (CD163–) (*P* < 0.05) in comparison with the LPS group, and only TLR-9 in the C-33A group induced an increase in comparison with all groups (*P* < 0.05).

**Figure 6:**
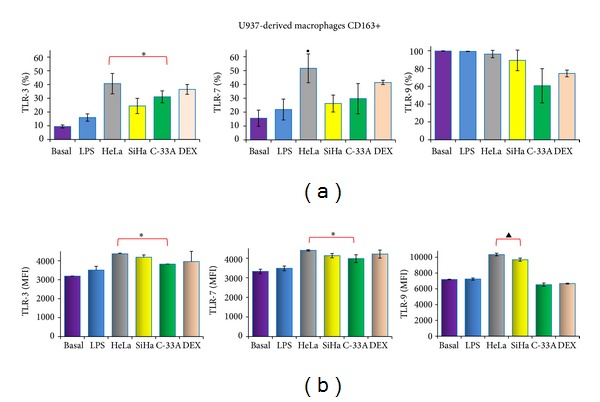
The supernatant of cervical cancer (CC) cells induces the expression of Toll-like receptor (TLR)-3, -7, and -9 in M2-like macrophages (CD163+). U937-derived macrophages were activated with lipopolysaccharides (LPS) and treated or not with supernatant of HeLa, SiHa, and C-33A cells or Dexamethasone (DEX) for 7 days. After that, the expression of TLR-3, -7, and -9 was analyzed in CD163+ cells induced by the supernatant of CC cells by flow cytometry (FC). Percentage of TLR-3, -7, and -9 expression on M2-like macrophages (CD163+) (a). Geometric mean fluorescence intensity (MFI) for TLR-3, -7, and -9 in the different experimental groups (b). Results are shown as % and MFI and represent the mean ± standard deviations (SD) of three independent experiments performed in triplicate. Statistical analysis, Student's *t*-test. *P < 0.05 Basal and LPS groups versus HeLa, SiHa, and C-33A groups. ^•^ *P* < 0.05 Basal and LPS versus HeLa group. ^▲^ *P* < 0.05 Basal and LPS versus HeLa and SiHa groups.

In Figure 6(a), we observed the expression of TLR-3 and TLR-7 in M2 macrophages (CD163+) which, in the LPS group, was 15.9 and 21.3%, respectively; additionally, the supernatant of HeLa cells induced a significant increase in these receptors (40.7 ± 7.4% for TLR-3 and 51.6 ± 10.4% for TLR-7, resp.; *P* < 0.05). C-33A only had this significant effect on TLR-3 expression (31.1 ± 4.3%; *P* < 0.05). TLR-9 expression was practically unchanged among the groups. In Figure 6(b), MFI is depicted for TLR-3, -7, and -9 in M2 macrophages (CD163+). CC lines significantly increased MFI of TLR-3 and TLR-7 in comparison with the LPS group, whereas, for TLR-9, only tumor cells infected with viruses HeLa and SiHa, induced an increase in MFI (*P* < 0.05). With the DEX group, we observed an increase in TLR-3 in comparison with the LPS group. Taken together, these results suggest that the supernatant from CC cell lines modified the expression of TLR.

## 4. Discussion

Macrophages have been considered important immune effectors cells against tumors; however, their relationships with cervical tumor cells are poorly understood. In general, two distinct polarization states have been described for macrophages: the M1 (or classically activated) state and the M2 (or alternatively activated) state [6, 23, 24]. Our results clearly show that the secretion of products by HeLa, SiHa, and C-33A cancer cells change the phenotype of macrophages from the M1 into the M2 phenotype. This is important because the presence of M1 or M2 can modify the prognosis of a patient with cancer [7, 15].

We worked with three different cell lines in order to reach greater proximity to clinical conditions. Two of these cell lines were infected with the most frequent HPV subtype, while the remaining cell line was not infected.

As reported in the literature, CD163 is a molecule expressed in monocyte-macrophage lineage [25]. Under normal conditions, this receptor is expressed in small amounts in the macrophages, and it is known that it is expressed in high amounts in M2 macrophages [8]. Our results show that CD163 is weakly expressed in U937-derived macrophages activated with LPS (M1 macrophages), but that its expression is strongly increased when these cells are treated with the supernatants of CC cell lines HeLa (HPV-18+), SiHa (HPV-16+), and, to a lesser degree, C-33A (HPV–). In agreement with this observation, Heusinkveld et al. reported that the supernatants of CC cell lines hampered the differentiation of monocytes into dendritic cells; instead, human monocytes cultured with supernatant were differentiated into M2 macrophages, expressing the CD163 marker [17]. Additionally, it was demonstrated that colon cancer cell supernatant induced, in THP-1-derived macrophages, an M2-like phenotype with an increase in CD163 expression [26]. Moreover, our results show that CD163 was not regulated positively by the immortalized nontumorigenic keratinocyte cell line HaCaT supernatant. In accordance with this observation, Linde et al., using an* in vivo* model of transfection, reported that HaCaT cells used as control do not induce M2 macrophages [27]. Our observations strongly suggest that the effect on CD163 expression is specific to CC cells, as is the existence of soluble factors secreted by cervical tumor cells, but not by nontumorigenic cells implicated in polarization into M2 macrophages. Additionally, in our study, we observed that, mainly, CC cells infected with HPV induced an increase in CD163 expression in U937-derived macrophages activated with LPS. Taken together, all of this information suggests that HPV plays a role in the transformation of M1 into M2, perhaps as amplifier, because noninfected C-33A virus cells are also able to change the phenotype of macrophages, despite the fact that this is effected in a less important manner.

It is very well known that there are cytokine profiles that allow the expression of either the M1 or the M2 phenotype. IL-6 and IL-13 are included within the M2 cytokine profile [9, 21]. The changes in this work from the M1 into the M2 phenotype, at least in part, may be considered as due to the IL-6 and IL-13 secreted by HeLa and SiHa cells. In accordance with our observations, it has been reported that IL-6 is expressed by cervical carcinoma cells infected with HPV and, on the other hand, IL-13 expression was demonstrated in cell lines derived from Hodgkin disease and pancreatic cell lines [28–32]. In addition, the fact that C-33A cells do not secrete IL-6 and IL-13 is in agreement with the idea that other factors are related with M2 phenotype generation.

In order to understand the results with HeLa and SiHa cells, it was reported that IL-6 blocks the production of TNF-*α* and induces the expression of IL-1 receptor antagonist and CD163 mRNA in monocytes and macrophages with M2 characteristics; likewise, IL-6 may facilitate the oncogenesis of human cervical cancer, positively regulating the expression of antiapoptotic genes such as* Mcl-1* [30, 33, 34]. On the other hand, IL-13 promotes an M2 macrophage phenotype [6, 8]. Based on this, we suggest that IL-6 and IL-13 were the key inducers in the expression of CD163 in macrophages by CC cell lines infected with HPV, HeLa, and SiHa cells. Another important consideration is that IL-4 is another key cytokine that promotes the M2 phenotype in macrophages [6, 9]; however, it was not found in any supernatant, suggesting either no detectable amounts of this cytokine, or the existence of other factors that influenced the change in phenotype into M2 macrophages.

The presence of IL-2 in several cancer cells has been published [35–38]; therefore, the importance of IL-2 lies in its being a growth factor of tumor cervical cell lines [35]. We observed that IL-2 is secreted by HeLa cells; its importance is that it may, at least indirectly, be implicated in the change of macrophage phenotype. However, in this regard and in apparent discrepancy, HeLa and C-33A cells secrete IL-12 with T_H_1 activity [6]. It is clear that our observations result in a complex balance among factors that induce M1 and others with opposite activity. This is important because, rather than the action of a cytokine, the final interaction and balance among these cytokines and other molecules released by CC cells can determine whether or not an activated macrophage undergoes change into its phenotype or activation status.

The profile of the secretion of cytokines is different in M1 and M2 macrophages [8, 9]. M1 macrophages produce high levels of proinflammatory cytokines such as TNF-*α*, IL-1*β*, and IL-6 and low quantities of IL-10, whereas M2 macrophages reverse this balance. We demonstrated that tumor cells, in addition to inducing CD163 expression in macrophages, are capable of influencing the profile of cytokine secretion by means of these cells. Our results show that U937-derived macrophages activated with LPS release high levels of TNF-*α*, IL-1*β*, and IL-6 cytokines. In addition, after the change of immunophenotype into M2 macrophages by the effect of HeLa, SiHa, and C-33A CC cells, M2 macrophages increase the release of IL-10, an anti-inflammatory cytokine, and decreased the secretion of proinflammatory cytokines. Additionally, NO production was reduced in U937-derived macrophages activated with LPS and treated with supernatants of CC cells, all of these suggesting a lesser antimicrobial and antitumor capacity, as well as lesser capacity for activating other cells of the innate immune system and of adaptive immunity. In this regard, it was documented that macrophages, through a direct effect of NO, can kill [39] or reduce the replication of viruses or can inhibit the growth of tumor cells through the inhibition of enzymes essential for tumor growth [40, 41].

The incubation of macrophages with supernatant from cervical tumor cells induced profound effects on TLR expression, because TLR-3, -7, and -9 are upregulated in M1 macrophages, mainly in the presence of supernatant from cells infected with HPV, such as HeLa and SiHa. This can indicate that, in response to molecules secreted by CC cells and in particular HPV+ cells, M1 macrophages upregulated the expression of TLR-3, -7, and -9, which could induce nuclear factor* kappa*-B (NF-*κ* *Β*) and Interferon regulatory transcription factor 3 (IRF3) pathway activation, facilitating host defense and tumor cell killing. In this respect, it is relevant that an increase was published in the expression viral nucleic-acid-sensing-TLR (TLR-3, -7, -8, and -9) in women who eliminate HPV-16 infections in comparison with women with persistent HPV-16 infections [13]. Additionally, when we analyzed TLR expression in M2 macrophages (CD163+), HeLa (HPV-18+) cells in these macrophages are the main inducers of upregulation of TLR-3 and TLR-7. This may infer that the macrophage is responding to the products secreted from CC cells by increasing TLR-mediated signaling in an attempt to establish compensatory mechanisms in order to reverse the process that induces a phenotype suppressor. However, this could be counterproductive or harmful, because it was demonstrated that TLR play a role in IL-10 induction through the activation of the extracellular signal-regulated kinase-Mitogen-activated protein kinase (ERK1/2 MAPK) pathway, which further triggered histone phosphorylation at the IL-10 promoter, causing enhanced IL-10 production in M2 macrophages [42]. Another point to be stressed is that the activation of nitric oxide synthase (iNOS) and cyclooxygenase-2 by TLR signaling resulted in an increase in IL-10 and vascular endothelial growth factor (VEGF) levels, as well as those of TGF-*β*, favoring the tumor microenvironment by mediating immune suppression [43].

Our results demonstrated that in CC, human cell lines HeLa (HPV-18+) and SiHa (HPV-16+) and, to a lesser degree, C-33A (HPV–), induce a change of immunophenotype in macrophages, from M1 macrophages into M2 macrophages, plus a change in the reduction of proinflammatory cytokines TNF-*α*, IL-1*β*, and IL-6, as well as decreased NO production and increased IL-10 production. The presence of the virus may play an important, but not a definitive, role in our observations.

## 5. Conclusions

Our results indicate that U937-derived macrophages activated with LPS (M1 macrophages) in response to CC cells respond by an increase in the expression of TLR, which facilitates the production of proinflammatory cytokines and NO, and that supernatants of HeLa, SiHa, and C-33A CC lines induce a change into M2-like macrophages characterized by CD163 upregulation, a decrease of proinflammatory cytokines and NO production, and an increase in IL-10.
